# Percutaneous coronary intervention versus coronary artery bypass grafting in patients with coronary heart disease and type 2 diabetes mellitus: Cumulative meta‐analysis

**DOI:** 10.1002/clc.23613

**Published:** 2021-06-05

**Authors:** Qiuping Xie, Jianguo Huang, Ke Zhu, Qing Chen

**Affiliations:** ^1^ Department of Cardiology Zhuzhou Central Hospital Zhuzhou China; ^2^ Department of Cardiology Liling Traditional Chinese Medicine Hospital Zhuzhou China

**Keywords:** coronary artery bypass graft, coronary heart disease, meta‐analysis, mortality, percutaneous coronary intervention, type 2 diabetes mellitus

## Abstract

Previous meta‐analyses showed that coronary artery bypass grafting (CABG) has lower all‐cause mortality than percutaneous coronary intervention (PCI) for the management of coronary heart disease (CHD), but the long‐term outcomes were not analyzed thoroughly in patients with type 2 diabetes mellitus (T2DM). To perform a meta‐analysis of randomized controlled trials (RCTs) to explore the long‐term effectiveness between CABG and PCI in patients with T2DM and study the temporal trends using a cumulative meta‐analysis. PubMed, Embase, Cochrane library, and Clinical Trials Registry for eligible RCTs published up to September 2020. The outcomes were all‐cause death, cardiac death, myocardial infarction, repeat revascularization, and stroke. Nine RCTs and 4566 patients were included. CABG resulted in better outcomes than PCI in terms of all‐cause death (RR = 1.41, 95%CI: 1.22–1.63, *p* < 0.001), cardiac death (RR = 1.56, 95%CI: 1.25–1.95, *p* < 0.001), and repeat revascularization (RR = 2.68, 95%CI: 1.86–3.85, *p* < 0.001), but with difference regarding the occurrence of myocardial infarction (RR = 1.20, 95%CI: 0.78–1.85, *p* = 0.414), while PCI was associated with better outcomes in terms of stroke occurrence (RR = 0.51, 95%CI: 0.34–0.77, *p* = 0.001). The cumulative meta‐analysis for all‐cause death showed that the differences between CABG and PCI started to be significant at 3 years of follow‐up, while the difference became significant at 5 years for cardiac death. In patients with CHD and T2DM, CABG results in better outcomes than PCI in terms of all‐cause death, cardiac mortality, and repeat revascularization, while PCI had better outcomes in terms of stroke. The differences are mainly observed over the long‐term follow‐up.

## 1 INTRODUCTION

Coronary heart disease (CHD) refers to myocardial dysfunction and organic lesions due to coronary artery stenosis and insufficient blood supply to heart areas. It has become the leading cause of death and disability worldwide.^1^ CHD affects 1.7% of the world population and is responsible for 9 million deaths each year.^2^ Type 2 diabetes mellitus (T2DM) is an independent risk factor of CHD.^3^ About 370 million people have T2DM in the world.^4^ The number of patients with coronary multi‐vessel disease and T2DM is increasing year by year.^3^

Coronary artery bypass grafting (CABG) refers to cardiac surgery during which a section of a blood vessel, either an artery or vein, is grafted to serve as a new conduit for improved blood flow to the heart.^5^ Percutaneous coronary intervention (PCI) is a non‐surgical procedure to improve coronary blood flow at the occlusion site via maneuvers performed through a coronary catheter, including balloon inflation, stent placement, and/or atherectomy.^6^ The two procedures have their pros and cons. CABG can provide long‐term control of the disease, but it is a significantly invasive procedure, and there is a risk of losing graft patency over time. PCI is a minimally‐invasive procedure, but in‐stent restenosis can occur, which can be prevented using a drug‐eluting stent (DES).^7^ PCI is associated with higher restenosis and revascularization rates than with CABG during revascularization in patients with coronary multi‐vessel disease complicated with T2DM; however, most data were obtained from the bare metal stent (BMS) era.^8^, ^9^, ^10^ Still, because of the chronic inflammatory and oxidative stress states observed in T2DM, guidelines recommend CABG for patients with T2DM and coronary multi‐vessel disease.^11^ With the advent of DES, some authors believed that was is associated with a better prognosis, significantly reducing in‐stent restenosis and revascularization rate after DES‐PCI,^8^, ^12^ but the advent of DES might have widened the gap with respect to major adverse cardiovascular events.^13^, ^14^ In multi‐vessel disease, it appears that CABG is associated with a better prognosis than PCI.^7^ Furthermore, patients with T2DM require antiplatelet therapy after revascularization, and the optimal strategy is different than in non‐T2DM patients,^15^ which could influence the conclusions of the various studies.

Previous meta‐analyses included randomized controlled trials (RCTs) and showed that CABG has lower all‐cause mortality than PCI,^7^, ^16^, ^17^, ^18^ but the long‐term outcomes were not analyzed thoroughly in patients with T2DM. Herein, we performed this updated meta‐analysis only based on RCTs study to explore the long‐term effectiveness between CABG and PCI in patients with CHD and T2DM. Moreover, a cumulative meta‐analysis was performed to study the temporal trends.

## 2 METHODS

### 2.1 Literature search

This meta‐analysis was performed according to the Preferred Reporting Items for Systematic Reviews and Meta‐Analyses (PRISMA) guidelines.^19^ A systematic search was performed in the PubMed, Embase, Cochrane library, and Clinical Trials Registry for available papers published up to September 2020. The terms ''PCI'' or ''percutaneous intervention'', ''CABG'' or ''coronary artery bypass grafting'', ''coronary disease'', and ''T2DM'' or ''type 2 diabetes mellitus'' were used. The reference lists from the retrieved studies were reviewed to identify any new eligible study. The eligibility criteria were (1) population: patients with coronary artery disease and T2DM, (2) intervention: CABG, (3) control: PCI, (4) outcome: all‐cause mortality, cardiovascular‐cause mortality, myocardial infarction (MI), repeat revascularization, and stroke, (5) study design: RCT, and (6) full text in English. Two authors (** and **) independently performed the literature search and selection.

### 2.2 Data extraction

Two authors (** and **) independently extracted the study characteristics (first author's name, trial's name, recruitment period, sampling size, age, sex, intervention arms, and follow‐up) and outcomes (if the study reported hazard ratios (HRs), the HRs and 95% confidence interval (CI) were extracted; if not, relative risks (RRs) and 95%CI were calculated based on the events in the two arms). When a paper reported multiple populations, the outcomes and population of interest were selected. Discrepancies were solved by discussion.

### 2.3 Quality of the evidence

The methodological quality of the RCTs was evaluated by two authors (** and **) independently with the quality assessment tool from the Cochrane manual. Discrepancies were solved by discussion.

### 2.4 Statistical analysis

RRs and corresponding 95% CIs were used to summarize the results. Statistical heterogeneity among the studies was calculated using Cochran's Q‐test and the I^2^ index. An I^2^ > 50% and Q‐test *p* < 0.10 indicated high heterogeneity. Considering the vast patient population that originated from different regions and the distinct variables that were adjusted for in different studies, the random‐effects model was used to avoid the risk of overestimating the associations. Besides, a cumulative meta‐analysis was conducted to study the temporal trends.^20^ The possible publication bias could not be evaluated by funnel plots and Egger's test because the number of studies included in each meta‐analysis was <10, in which case the funnel plots and Egger's test could yield misleading results. All analyses were performed using STATA SE 14.0 (StataCorp, College Station, TX).

## 3 RESULTS

### 3.1 Characteristics of the studies

Figure S1 presents the study selection process. The initial search produced 1090 records. After removing the duplicates, 905 records were screened, and 776 were excluded. Then, 129 full‐text articles were assessed for eligibility and 120 were excluded (conference abstract and review, *n* = 21; study aim/design, *n* = 40; population, *n* = 14; intervention, *n* = 11; outcomes, *n* = 29; non‐English, *n* = 5).

Finally, nine RCTs were included^14^, ^21^, ^22^, ^23^, ^24^, ^25^, ^26^, ^27^, ^28^, ^29^, ^30^ (Table S1). There were 2266 patients in the CABG arm and 2300 in the PCI arm. Figure S2 shows that all nine trials carried a high risk of bias in at least one category.

### 3.2 CABG, PCI, and all‐cause death

**FIGURE 1:**
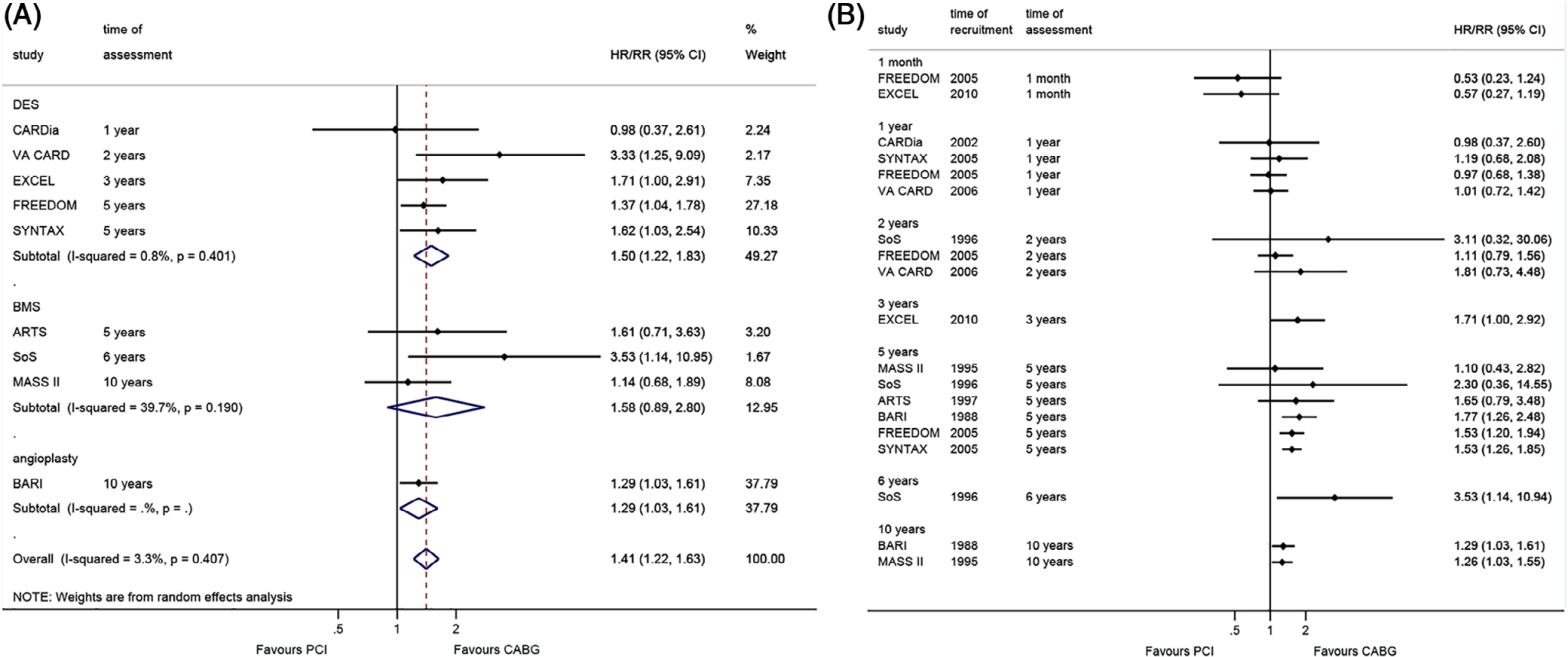
Effect of coronary artery bypass graft and percutaneous coronary intervention on all‐cause death. (A). within the longest follow‐up. (B). cumulative meta‐analysis

The meta‐analysis performed at the longest follow‐up shows that CABG was associated with better survival than PCI (RR = 1.41, 95%CI: 1.22–1.63, *p* < 0.001; I^2^ = 3.3%, P_heterogeneity_ = 0.407) (Figure 1(A)). When analyzing by the type of stent, CABG was still associated with a better survival than DES PCI (RR = 1.50, 95%CI: 1.22–1.83, *p* < 0.001; I^2^ = 0.8%, P_heterogeneity_ = 0.401) and angioplasty (RR = 1.29, 95%CI: 1.03–1.61, *p* = 0.025), but there was no difference with bare‐metal stent (BMS) PCI (RR = 1.58, 95%CI: 0.89–2.80, *p* = 0.119; I^2^ = 39.7%, P_heterogeneity_ = 0.190) (Figure 1(A)). The cumulative meta‐analysis shows that the trials reporting outcomes at 1 month, 1 year, and 3 years showed no significant differences between CABG and PCI. Still, the differences started to be significant at 3 years in one trial, 5 years in three out of six trials, at 6 years in one trial, and 10 years in two trials (Figure 1(B)).

### 3.3 CABG, PCI, and cardiac death

**FIGURE 2:**
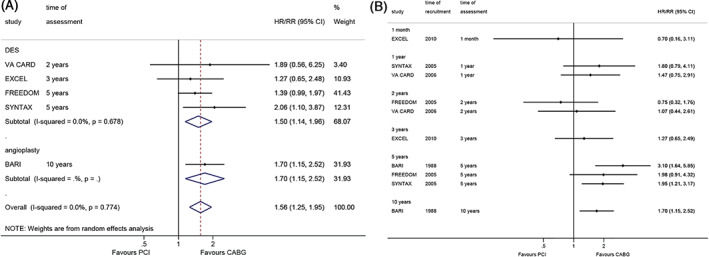
Effect of coronary artery bypass graft and percutaneous coronary intervention on cardiac death. (A). within the longest follow‐up. (B). cumulative meta‐analysis

Figure 2(A) shows that CABG led to better survival than PCI in cardiac death (RR = 1.56, 95%CI: 1.25–1.95, *p* < 0.001; I^2^ = 0%, P_heterogeneity_ = 0.774). The same associations were observed when comparing CABG with DES PCI (RR = 1.50, 95%CI: 1.14–1.96, *p* = 0.003; I^2^ = 0%, P_heterogeneity_ = 0.678) and angioplasty (RR = 1.70, 95%CI: 1.15–2.52, *p* = 0.008). The cumulative meta‐analysis showed that the differences were not significant at 1 month and 1, 2, and 3 years, but it was significant at 5 years in two of three studies and 10 years in one study (Figure 2(B)).

### 3.4 CABG, PCI, and MI

**FIGURE 3:**
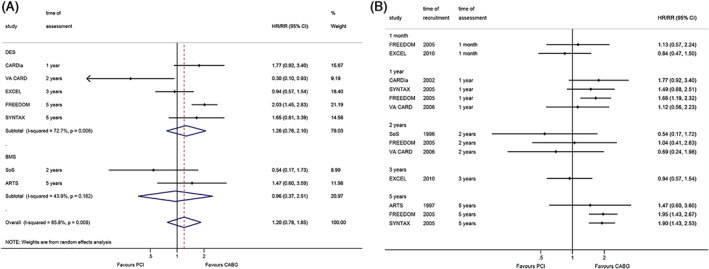
Effect of coronary artery bypass graft and percutaneous coronary intervention on myocardial infarction. (A). within the longest follow‐up. (B). cumulative meta‐analysis

There were no differences between CABG and PCI regarding the occurrence of MI (RR = 1.20, 95%CI: 0.78–1.85, *p* = 0.414; I^2^ = 65.8%, P_heterogeneity_ = 0.008), and when considering DES PCI (RR = 1.26, 95%CI: 0.76–2.10, *p* = 0.367; I^2^ = 72.7%, P_heterogeneity_ = 0.006) and BMS PCI (RR = 0.96, 95%CI: 0.37–2.51, *p* = 0.931; I^2^ = 43.9%, P_heterogeneity_ = 0.182) (Figure 3(A)). The cumulative meta‐analysis showed that only two of three studies reported benefits from CABG at 5 years.

### 3.5 CABG, PCI, and repeat vascularization

**FIGURE 4:**
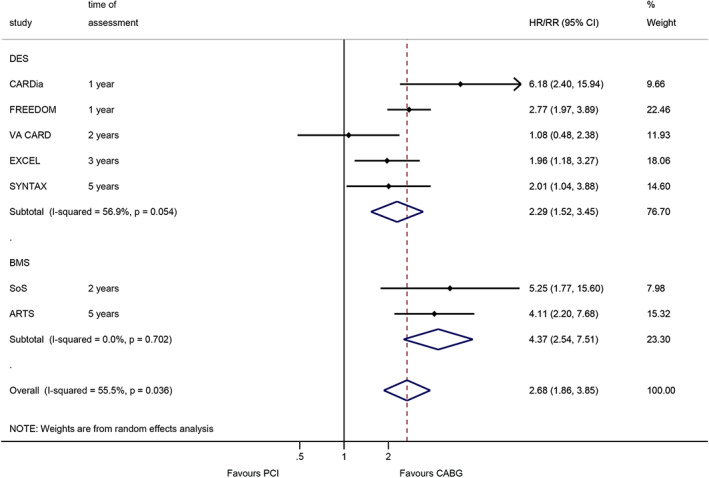
Effect of coronary artery bypass graft and percutaneous coronary intervention on repeat revascularization (within the longest follow‐up)

The meta‐analysis performed at the longest follow‐up shows that CABG was associated with a lower occurrence of repeat revascularization (RR = 2.68, 95%CI: 1.86–3.85, *p* < 0.001; I^2^ = 55.5%, P_heterogeneity_ = 0.036) (Figure 4). When analyzing by the type of stent, CABG was still associated with a better survival than DES PCI (RR = 2.29, 95%CI: 1.52–3.45, *p* < 0.001; I^2^ = 56.9%, P_heterogeneity_ = 0.054) and BMS PCI (RR = 4.37, 95%CI: 2.54–7.51, *p* < 0.001; I^2^ = 0%, P_heterogeneity_ = 0.702) (Figure 4).

### 3.6 CABG, PCI, and stroke

**FIGURE 5:**
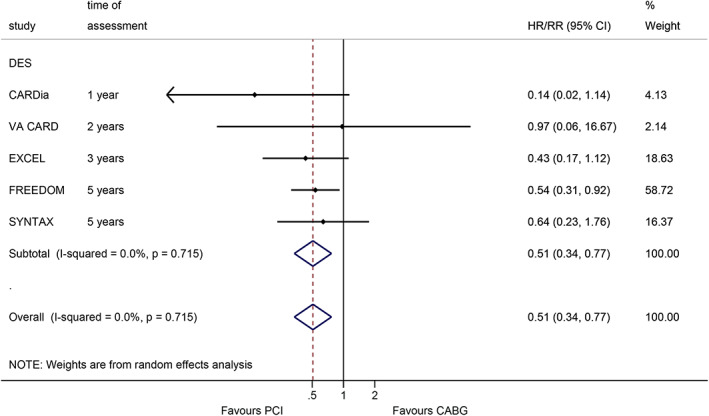
Effect of coronary artery bypass graft and percutaneous coronary intervention on stroke (within the longest follow‐up)

At the longest follow‐up, DES PCI was associated with a lower occurrence of stroke (RR = 0.51, 95%CI: 0.34–0.77, *p* = 0.001; I^2^ = 0%, P_heterogeneity_ = 0.715) (Figure 5).

## 4 DISCUSSION

Previous meta‐analyses showed that CABG has lower all‐cause mortality than PCI for the management of CHD,^7^, ^16^, ^17^, ^18^, ^31^, ^32^ but the long‐term outcomes were not analyzed thoroughly in patients with T2DM. Therefore, this meta‐analysis of RCTs to explore the long‐term effectiveness between CABG and PCI in patients T2DM and to study the temporal trends using a cumulative meta‐analysis. The results show that in patients with CHD and T2DM, CABG results in better outcomes than PCI in terms of all‐cause death, cardiac mortality, and repeat revascularization, while PCI had better outcomes in terms of stroke. The differences are mainly observed over the long‐term follow‐up.

In the pre‐DES era, previous meta‐analyses did not found significant differences in long‐term survival between PCI and CABG in diabetic patients, and that was also recently observed in both cardiac and non‐cardiac mortality where better survival with CABG was seen only in the DES era.^13^, ^33^, ^34^, ^35^, ^36^ Still, multiple previous meta‐analyses suggested better outcomes of CABG than PCI.^7^, ^16^, ^17^, ^18^, ^31^, ^32^ Spadaccio et al^7^ showed that CABG had better outcomes than DES PCI regarding MI, stroke, and repeat revascularization. Athappan et al^16^ performed a meta‐analysis of three RCTs and showed that the rates of major adverse cardio‐cerebrovascular events (MACCES) and repeat revascularization were high after PCI. Ali et al^17^ showed that CABG had advantages over PCI in MACCES, repeat vascularization, and MI, while the stroke risk was lower with PCI. Ahmad et al^18^ reported no differences in any outcomes between PCI and CABG. Gallo et al^31^ showed no differences between CABG and PCI in 5‐year mortality, while CABG was associated with a higher risk of stroke at 1 and 12 months, while PCI was associated with higher risks of MI and repeat revascularization at 5 years. Zhai et al^32^ performed a meta‐analysis of RCTs and observational studies in diabetic patients. They showed that CABG was superior to PCI in diabetic patients but that the risk of stroke was higher. Globally, those previous meta‐analyses performed in various patient populations with CHD and including various study designs support the present meta‐analysis that included only diabetic patients and RCTs. The combined results suggest better CABG outcomes regarding mortality, MI, and repeat vascularization, but a higher risk of stroke. This is supported by a 12‐year follow‐up study from the MAIN COMPARE registry.^37^ This study could not examine the causes of this increased risk of stroke, but it could coincide with antiplatelet therapy discontinuation after 1 year.^38^, ^39^ This could also be due to cerebral embolism due to manipulating aortic atherosclerotic plaques during CABG^40^, ^41^ and cerebral hypoperfusion during CABG.^42^, ^43^

The incidence of CHD in diabetic patients is 2–4 times higher than that in non‐diabetic individuals, and the odds of multi‐vessel disease and a higher degree of disease are higher than in non‐diabetics.^44^, ^45^, ^46^ Many studies showed that CABG seems to be a better choice than PCI in elderly patients with CHD and T2DM, especially in the case of multi‐vessel CHD.^47^, ^48^ For example, previous reports regarding the data on heart disease (diabetic coronary revascularization) trials and 5‐year results showed that the incidence of MACCES associated with PCI was significantly higher than that of CABG patients with T2DM.^49^ Bundhun et al.^50^ stated that MACCES, as well as repeated revascularization, were significantly lower in the CABG group than those in the PCI group, supporting the present study, albeit MACCES could not be analyzed here because of too vast differences in the definitions of MACCES among the included studies. According to Kappetein et al.,^12^ PCI resulted in a high incidence of MACCES and repeated vascular reconstruction within 5 years in diabetic and non‐diabetic patients. Therefore, although PCI is a potential treatment, CABG should be considered as the first choice for vascular reconstruction in patients with more complex anatomical diseases, especially in patients with diabetes mellitus.

In patients whose 3‐5‐year health status exceeds the survival period (recognizing the 1‐year health status of CABG), the perioperative risk associated with CABG might require attention (i.e., in elderly patients). In this study, the cumulative meta‐analysis approach suggests that the differences between CABG and PCI became significant at 3 years. This contradicts a meta‐analysis by Athappan et al.,^16^ who showed that the differences between CABG and PCI disappeared after 5 years. Those conflicting results could be due to the different studies being included and the meta‐analysis method (cumulative meta‐analysis, in the present study). Hence, carefully weighing the operative risks with the expected survival is essential to select the best approach.

There are some limitations that must be considered. First, heterogeneity was observed for many analyses, and the random‐effect model was used in all analyses because of the heterogeneity of the included patient populations. This avoids over‐estimating the associations but results in weaker associations than the actual associations. Besides, heterogeneity might be because positive results have a higher chance of publication. In addition, limiting the articles to English could lead to the omission of valuable results that could tip the balance. Second, studies from more countries should be analyzed since there are disparities among countries regarding economy and guidelines. Third, due to heterogeneity in definitions of composite outcomes such as MACCE, the present meta‐analysis did not analysis these data. Finally, no patient‐level data were available and could be analyzed. Further research on composite outcomes over long‐term follow‐up with larger sample sizes is warranted in the future.

In conclusion, in patients with CHD and T2DM, CABG results in better outcomes than PCI in terms of all‐cause death, cardiac mortality, and repeat revascularization, while PCI had better outcomes in terms of stroke. The differences are mainly observed over the long‐term follow‐up and when comparing with patients with a DES. This study also suggests that a BMS is no worse than a DES in diabetics. Future studies could examine outcomes like MACCE, surgical complications, in‐hospital mortality, quality of life, and overall costs of CABG vs. PCI in patients with CHD and T2DM.

## CONFLICT OF INTEREST

The authors declare no potential conflict of interest.

## Supporting information


**Table S1** Basic characteristics of eligible studies.

**Figure S1** PRISMA flowchart of study selection.

**Figure S2** Methodological quality of RCTs included in the meta‐analysis.

## Data Availability

The data set supporting the results of this article are included within the article and supplementary materials.

## References

[1] Naito R , Miyauchi K , Konishi H , Tsuboi S , Ogita M , et al. Comparing mortality between coronary artery bypass grafting and percutaneous coronary intervention with drug‐eluting stents in elderly with diabetes and multivessel coronary disease. Heart Vessels. 2016;31(9):1424‐1429. 26412228 10.1007/s00380-015-0746-1 PMC5010596

[2] Khan MA , Hashim MJ , Mustafa H , Baniyas MY , Al Suwaidi S , et al. Global epidemiology of ischemic heart disease: results from the global burden of disease study. Cureus. 2020;12(7):e9349. 32742886 10.7759/cureus.9349 PMC7384703

[3] Barnett KN , Ogston SA , McMurdo ME , Morris AD , Evans JM . A 12‐year follow‐up study of all‐cause and cardiovascular mortality among 10,532 people newly diagnosed with type 2 diabetes in Tayside, Scotland. Diabetes Med. 2010;27(10):1124‐1129. 10.1111/j.1464-5491.2010.03075.x 20854379

[4] Chatterjee S , Khunti K , Davies MJ . Type 2 diabetes. Lancet. 2017;389(10085):2239‐2251. 28190580 10.1016/S0140-6736(17)30058-2

[5] Diodato M , Chedrawy EG . Coronary artery bypass graft surgery: the past, present, and future of myocardial revascularisation. Surg Res Pract. 2014;2014:726158. 25374960 10.1155/2014/726158 PMC4208586

[6] Keeley EC , Hillis LD . Primary PCI for myocardial infarction with ST‐segment elevation. N Engl J Med. 2007;356(1):47‐54. 17202455 10.1056/NEJMct063503

[7] Spadaccio C , Benedetto U . Coronary artery bypass grafting (CABG) vs. percutaneous coronary intervention (PCI) in the treatment of multivessel coronary disease: quo vadis? ‐a review of the evidences on coronary artery disease. Ann Cardiothorac Surg. 2018;7(4):506‐515. 30094215 10.21037/acs.2018.05.17 PMC6082779

[8] CABG better than PCI for smokers and those with diabetes, PAD, or heart failure. BMJ. 2013;346:f2534. 23616375 10.1136/bmj.f2534

[9] Mukherjee D . Percutaneous coronary intervention versus coronary artery bypass grafting in diabetic patients. Cardiol Clin. 2005;23(2):185‐191. 15694746 10.1016/j.ccl.2004.06.003

[10] Roffi M , Brandle M , Robbins MA , Mukherjee D . Current perspectives on coronary revascularization in the diabetic patient. Indian Heart J. 2007;59(2):124‐136. 19122245

[11] Patel MR , Calhoon JH , Dehmer GJ , Grantham JA , Maddox TM , et al. ACC/AATS/AHA/ASE/ASNC/SCAI/SCCT/STS 2017 appropriate use criteria for coronary revascularization in patients with stable ischemic heart disease: a report of the American College of Cardiology Appropriate use Criteria Task Force, American Association for Thoracic Surgery, American Heart Association, American Society of Echocardiography, American Society of Nuclear Cardiology, Society for Cardiovascular Angiography and Interventions, Society of Cardiovascular Computed Tomography, and Society of Thoracic Surgeons. J Am Coll Cardiol. 2017;69(17):2212‐2241. 28291663 10.1016/j.jacc.2017.02.001

[12] Kappetein AP , Head SJ . CABG or PCI for revascularisation in patients with diabetes? Lancet Diabetes Endocrinol. 2013;1(4):266‐268. 24622407 10.1016/S2213-8587(13)70114-1

[13] Gaudino M , Hameed I , Farkouh ME , Rahouma M , Naik A , et al. Overall and cause‐specific mortality in randomized clinical trials comparing percutaneous interventions with coronary bypass surgery: a meta‐analysis. JAMA Intern Med. 2020;180(12):1638‐1646. 33044497 10.1001/jamainternmed.2020.4748 PMC7551235

[14] Kapur A , Hall RJ , Malik IS , Qureshi AC , Butts J , et al. Randomized comparison of percutaneous coronary intervention with coronary artery bypass grafting in diabetic patients. 1‐year results of the CARDia (coronary artery revascularization in diabetes) trial. J Am Coll Cardiol. 2010;55(5):432‐440. 20117456 10.1016/j.jacc.2009.10.014

[15] Sharma A , Garg A , Elmariah S , Drachman D , Obiagwu C , et al. Duration of dual antiplatelet therapy following drug‐eluting stent implantation in diabetic and non‐diabetic patients: a systematic review and meta‐analysis of randomized controlled trials. Prog Cardiovasc Dis. 2018;60(4–5):500‐507. 29277295 10.1016/j.pcad.2017.12.003

[16] Athappan G , Vinodhkumaradithyaa A , Srinivasan M , Jeyaseelan L , Ponniah T . Meta‐analysis of 5‐year outcomes of CABG vs PCI with stenting in patients with multivessel disease. Minerva Cardioangiol. 2008;56(5):453‐460. 18813180

[17] Ali WE , Vaidya SR , Ejeh SU , Okoroafor KU . Meta‐analysis study comparing percutaneous coronary intervention/drug eluting stent versus coronary artery bypass surgery of unprotected left main coronary artery disease: clinical outcomes during short‐term versus long‐term (> 1 year) follow‐up. Medicine. 2018;97(7):e9909. 29443766 10.1097/MD.0000000000009909 PMC5839846

[18] Ahmad Y , Howard JP , Arnold AD , Cook CM , Prasad M , et al. Mortality after drug‐eluting stents vs. coronary artery bypass grafting for left main coronary artery disease: a meta‐analysis of randomized controlled trials. Eur Heart J. 2020;41(34):3228‐3235. 32118272 10.1093/eurheartj/ehaa135 PMC7557472

[19] Selcuk AA . A guide for systematic reviews: PRISMA. Turk Arch Otorhinolaryngol. 2019;57(1):57‐58. 31049257 10.5152/tao.2019.4058 PMC6461330

[20] Leimu R , Koricheva J . Cumulative meta‐analysis: a new tool for detection of temporal trends and publication bias in ecology. Proc Biol Sci. 2004;271(1551):1961‐1966. 15347521 10.1098/rspb.2004.2828 PMC1691819

[21] Detre KM , Guo P , Holubkov R , Califf RM , Sopko G , et al. Coronary revascularization in diabetic patients: a comparison of the randomized and observational components of the Aypass angioplasty revascularization investigation (BARI). Circulation. 1999;99(5):633‐640. 9950660 10.1161/01.cir.99.5.633

[22] Soares PR , Hueb WA , Lemos PA , Lopes N , Martinez EE , et al. Coronary revascularization (surgical or percutaneous) decreases mortality after the first year in diabetic subjects but not in nondiabetic subjects with multivessel disease: an analysis from the medicine, angioplasty, or surgery study (MASS II). Circulation. 2006;114(1 Suppl):I420‐I424. 16820611 10.1161/CIRCULATIONAHA.105.000679

[23] Lima EG , Hueb W , Garcia RM , Pereira AC , Soares PR , et al. Impact of diabetes on 10‐year outcomes of patients with multivessel coronary artery disease in the medicine, angioplasty, or surgery study II (MASS II) trial. Am Heart J. 2013;166(2):250‐257. 23895807 10.1016/j.ahj.2013.04.017

[24] Serruys PW , Ong AT , van Herwerden LA , Sousa JE , Jatene A , et al. Five‐year outcomes after coronary stenting versus bypass surgery for the treatment of multivessel disease: the final analysis of the arterial revascularization therapies study (ARTS) randomized trial. J Am Coll Cardiol. 2005;46(4):575‐581. 16098418 10.1016/j.jacc.2004.12.082

[25] Banning AP , Westaby S , Morice MC , Kappetein AP , Mohr FW , et al. Diabetic and nondiabetic patients with left main and/or 3‐vessel coronary artery disease: comparison of outcomes with cardiac surgery and paclitaxel‐eluting stents. J Am Coll Cardiol. 2010;55(11):1067‐1075. 20079596 10.1016/j.jacc.2009.09.057

[26] Kappetein AP , Head SJ , Morice MC , Banning AP , Serruys PW , et al. Treatment of complex coronary artery disease in patients with diabetes: 5‐year results comparing outcomes of bypass surgery and percutaneous coronary intervention in the SYNTAX trial. Eur J Cardio‐Thorac Surg. 2013;43(5):1006‐1013. 10.1093/ejcts/ezt017 23413014

[27] Farkouh ME , Domanski M , Sleeper LA , Siami FS , Dangas G , et al. Strategies for multivessel revascularization in patients with diabetes. N Engl J Med. 2012;367(25):2375‐2384. 23121323 10.1056/NEJMoa1211585

[28] Milojevic M , Serruys PW , Sabik JF 3rd , Kandzari DE , Schampaert E , et al. Bypass surgery or stenting for left Main coronary artery disease in patients with diabetes. J Am Coll Cardiol. 2019;73(13):1616‐1628. 30947913 10.1016/j.jacc.2019.01.037

[29] Kamalesh M , Sharp TG , Tang XC , Shunk K , Ward HB , et al. Percutaneous coronary intervention versus coronary bypass surgery in United States veterans with diabetes. J Am Coll Cardiol. 2013;61(8):808‐816. 23428214 10.1016/j.jacc.2012.11.044

[30] Booth J , Clayton T , Pepper J , Nugara F , Flather M , et al. Randomized, controlled trial of coronary artery bypass surgery versus percutaneous coronary intervention in patients with multivessel coronary artery disease: six‐year follow‐up from the stent or surgery trial (SoS). Circulation. 2008;118(4):381‐388. 18606919 10.1161/CIRCULATIONAHA.107.739144

[31] Gallo M , Blitzer D , Laforgia PL , Doulamis IP , Perrin N , et al. Percutaneous coronary intervention versus coronary artery bypass graft for left main coronary artery disease: a meta‐analysis. J Thorac Cardiovasc Surg. 2020. 10.1016/j.jtcvs.2020.04.010 32499076

[32] Zhai C , Cong H , Hou K , Hu Y , Zhang J , Zhang Y . Clinical outcome comparison of percutaneous coronary intervention and bypass surgery in diabetic patients with coronary artery disease: a meta‐analysis of randomized controlled trials and observational studies. Diabetol Metab Syndr. 2019;11:110. 31890044 10.1186/s13098-019-0506-y PMC6923849

[33] Assimes T , Hlatky MA . Cardiac outcomes occurred more frequently with PCI than CABG or medical therapy in coronary artery disease. ACP J Club. 2004;141(3):57. 15518441 10.7326/ACPJC-2004-141-3-057

[34] Flather M , Rhee JW , Boothroyd DB , Boersma E , Brooks MM , et al. The effect of age on outcomes of coronary artery bypass surgery compared with balloon angioplasty or bare‐metal stent implantation among patients with multivessel coronary disease. A collaborative analysis of individual patient data from 10 randomized trials. J Am Coll Cardiol. 2012;60(21):2150‐2157. 23153843 10.1016/j.jacc.2012.08.982

[35] Hlatky MA , Boothroyd DB , Bravata DM , Boersma E , Booth J , et al. Coronary artery bypass surgery compared with percutaneous coronary interventions for multivessel disease: a collaborative analysis of individual patient data from ten randomised trials. Lancet. 2009;373(9670):1190‐1197. 19303634 10.1016/S0140-6736(09)60552-3

[36] Daemen J , Boersma E , Flather M , Booth J , Stables R , et al. Long‐term safety and efficacy of percutaneous coronary intervention with stenting and coronary artery bypass surgery for multivessel coronary artery disease: a meta‐analysis with 5‐year patient‐level data from the ARTS, ERACI‐II, MASS‐II, and SoS trials. Circulation. 2008;118(11):1146‐1154. 18725490 10.1161/CIRCULATIONAHA.107.752147

[37] Hyun J , Kim JH , Jeong Y , Choe K , Lee J , et al. Long‐term outcomes after PCI or CABG for left main coronary artery disease according to lesion location. JACC Cardiovasc Interv. 2020;13(24):2825‐2836. 33357520 10.1016/j.jcin.2020.08.021

[38] Authors/Task Force M , Piepoli MF , Hoes AW , Agewall S , Albus C , et al. 2016 European guidelines on cardiovascular disease prevention in clinical practice: the sixth joint task force of the European Society of Cardiology and Other Societies on cardiovascular disease prevention in clinical practice (constituted by representatives of 10 societies and by invited experts): developed with the special contribution of the European Association for Cardiovascular Prevention & rehabilitation (EACPR). Eur J Prev Cardiol. 2016;23(11):NP1‐NP96. 27353126 10.1177/2047487316653709

[39] Hillis LD , Smith PK , Anderson JL , Bittl JA , Bridges CR , et al. 2011 ACCF/AHA guideline for coronary artery bypass graft surgery: a report of the American College of Cardiology Foundation/American Heart Association task force on practice guidelines. Circulation. 2011;124(23):e652‐e735. 22064599 10.1161/CIR.0b013e31823c074e

[40] Palmerini T , Biondi‐Zoccai G , Reggiani LB , Sangiorgi D , Alessi L , et al. Risk of stroke with coronary artery bypass graft surgery compared with percutaneous coronary intervention. J Am Coll Cardiol. 2012;60(9):798‐805. 22917004 10.1016/j.jacc.2011.10.912

[41] Roach GW , Kanchuger M , Mangano CM , Newman M , Nussmeier N , et al. Adverse cerebral outcomes after coronary bypass surgery. Multicenter study of perioperative ischemia research group and the Ischemia Research and Education Foundation investigators. N Engl J Med. 1996;335(25):1857‐1863. 8948560 10.1056/NEJM199612193352501

[42] Davila‐Roman VG , Murphy SF , Nickerson NJ , Kouchoukos NT , Schechtman KB , Barzilai B . Atherosclerosis of the ascending aorta is an independent predictor of long‐term neurologic events and mortality. J Am Coll Cardiol. 1999;33(5):1308‐1316. 10193732 10.1016/s0735-1097(99)00034-0

[43] Bergman P , van der Linden J . Atherosclerosis of the ascending aorta as a major determinant of the outcome of cardiac surgery. Nat Clin Pract Cardiovasc Med. 2005;2(5):246‐251. 16265508 10.1038/ncpcardio0192

[44] Harrington RA . ACP journal Club. PCI using drug‐eluting stents had higher mortality than CABG in diabetes and multivessel CAD. Ann Intern Med. 2013;158(6):Jc8. 10.7326/0003-4819-158-6-201303190-02008 23552875

[45] Hallberg V , Palomaki A , Lahtela J , Voutilainen S , Tarkka M , Kataja M . Associations of metabolic syndrome and diabetes mellitus with 16‐year survival after CABG. Cardiovasc Diabetol. 2014;13:25. 24447406 10.1186/1475-2840-13-25 PMC3914357

[46] Greason KL , Schaff HV . Coronary artery bypass graft surgery (CABG) for patients with diabetes and multivessel coronary artery disease: identifying patients who would benefit with CABG and understanding the potential mechanisms involved. Coron Artery Dis. 2010;21(7):402‐406. 20706113 10.1097/MCA.0b013e32833bfde3

[47] Buckley E , Kearney P . The prevalence of diabetes mellitus in patients with multivessel disease undergoing PCI and CABG before and after the freedom trial. Have the trial findings impacted practice. Heart. 2015;101(5):27.

[48] Baradari AG , Emami Zeydi A , Aarabi M , Ghafari R . Metformin as an adjunct to insulin for glycemic control in patients with type 2 diabetes after CABG surgery: a randomized double blind clinical trial. Pak J Biol Sci. 2011;14(23):1047‐1054. 22590838 10.3923/pjbs.2011.1047.1054

[49] Agirbasli M . Influence of diabetes on CABG patency: targeting functional status after CABG in patients with DM. J Am Coll Cardiol. 2017;70(20):2604. 29145959 10.1016/j.jacc.2017.07.801

[50] Aggarwal B , Goel SS , Sabik JF , Shishehbor MH . The FREEDOM trial: in appropriate patients with diabetes and multivessel coronary artery disease, CABG beats PCI. Cleve Clin J Med. 2013;80(8):515‐523. 23908108 10.3949/ccjm.80a.13030

