# Supplementary material for: Percutaneous coronary intervention versus coronary artery bypass grafting in patients with coronary heart disease and type 2 diabetes mellitus: Cumulative meta‐analysis
Source: Clin Cardiol. 2021 Jun 5;44(7):899–906. doi: 10.1002/clc.23613 (PMC8259162; doi:10.1002/clc.23613)
Supplement: Supplementary file 1 — Table S1 Basic characteristics of eligible studies. [file CLC-44-899-s001.docx]

**Table S1.** Literature search and study characteristics.

| Study | Author, year | Patients in the CABG group | Patients in the PCI group | PCI-eluting stent | Age (years) | | Males | | Follow-up |
| --- | --- | --- | --- | --- | --- | --- | --- | --- | --- |
|  |  |  |  |  | CABG | PCI | CABG | PCI |  |
| BARI | Detre, 1999 ^29^ | 173 | 170 | NR | 62.3 | | 57% | | 5, 10 years |
| MASS II | Soares, 2006 ^30^ | 56 | 59 | BMS and DES | 60±8 | 61±10 | 67% | 54% | 5 years |
|  | Lima, 2013 ^31^ | 80 | 64 | BMS and DES | 59±8 | 61±9 | 72% | 56% | 10 years |
| ARTS-I | Serruys, 2005 ^32^ | 96 | 112 | BMS | 62.6 | 62.5 | 66% | 82% | 5 years |
| SYNTAX | Banning, 2010 ^33^ | 221 | 231 | DES | 65.4±9.2 | | 71% | | 1 years |
|  | Kappetein, 2013 ^34^ |  |  |  |  |  |  |  | 5 years |
| CARDia | Kapur, 2010 ^35^ | 254 | 256 | BMS or DES | 63.6±9.1 | 64.3±8.5 | 77.9% | 70.7% | 1 years |
| FREEDOM | Farkouh, 2012 ^36^ | 947 | 953 | DES | 63.1±9.2 | 63.2±8.9 | 69.5% | 73.2% | 1m, 1,2 ,5 years |
| EXCEL | Milojevic, 2019 ^37^ | 268 | 286 | EES | 66.5±9.2 | | 74.% | | 1m, 3years |
| VA CARD | Kamalesh, 2013 ^38^ | 97 | 101 | DES | 62.1±7.4 | 62.7±7.1 | 99% | 99.% | 1, 2years |
| SoS | Booth, 2008 ^39^ | 74 | 68 | BMS | 62 | | 79% | | 2, 5, 6 years |

CABG: coronary artery bypass graft; PCI: percutaneous coronary intervention; NR: not reported; BMS: bare-metal stent; DES: drug-eluting stent
