# Supplementary figures and images for: Percutaneous coronary intervention versus coronary artery bypass grafting in patients with coronary heart disease and type 2 diabetes mellitus: Cumulative meta‐analysis
Source: Clin Cardiol. 2021 Jun 5;44(7):899–906. doi: 10.1002/clc.23613 (PMC8259162; doi:10.1002/clc.23613)

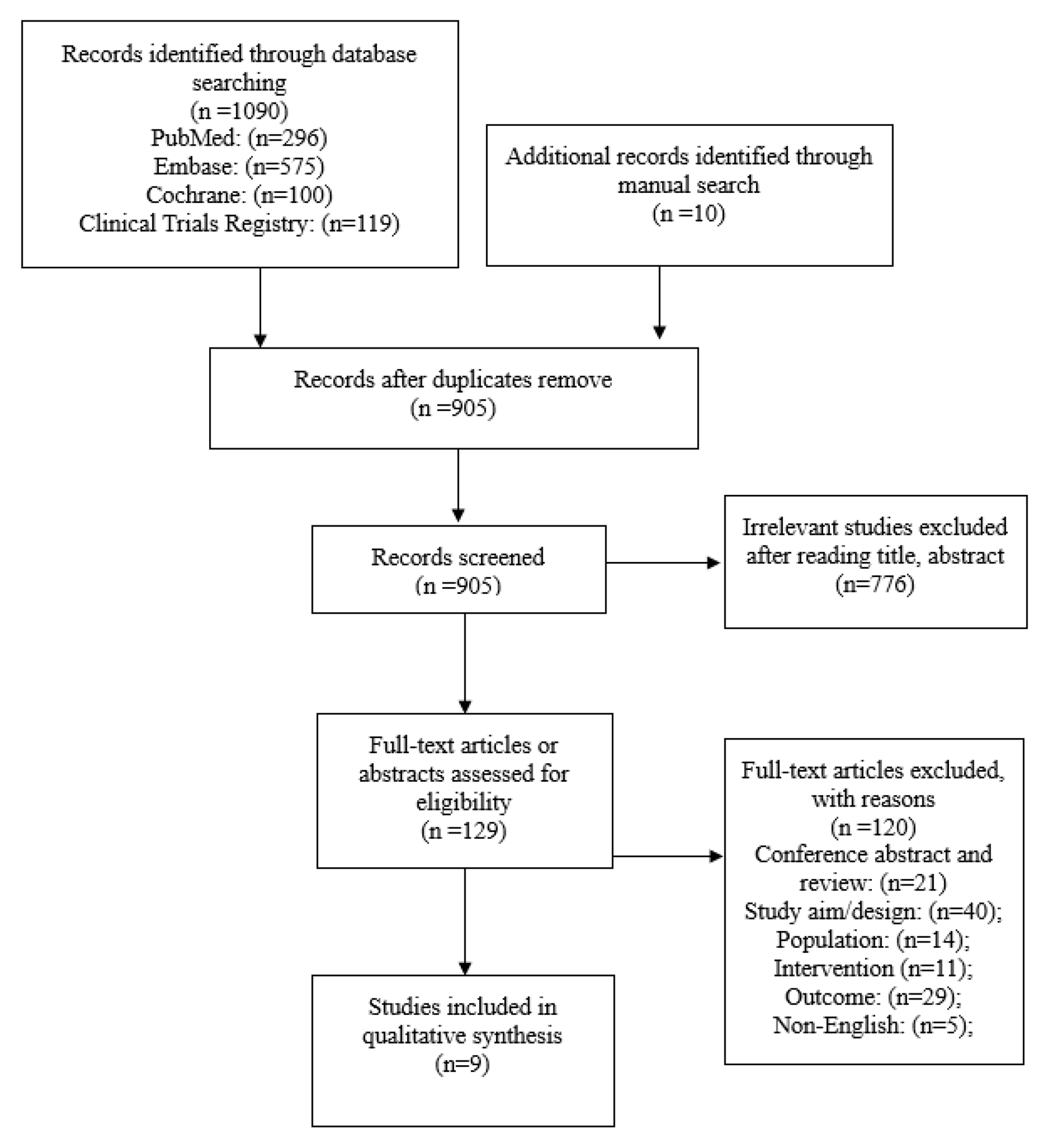

Supplement: Supplementary file 2 — Figure S1 PRISMA flowchart of study selection. [file CLC-44-899-s002.tif]

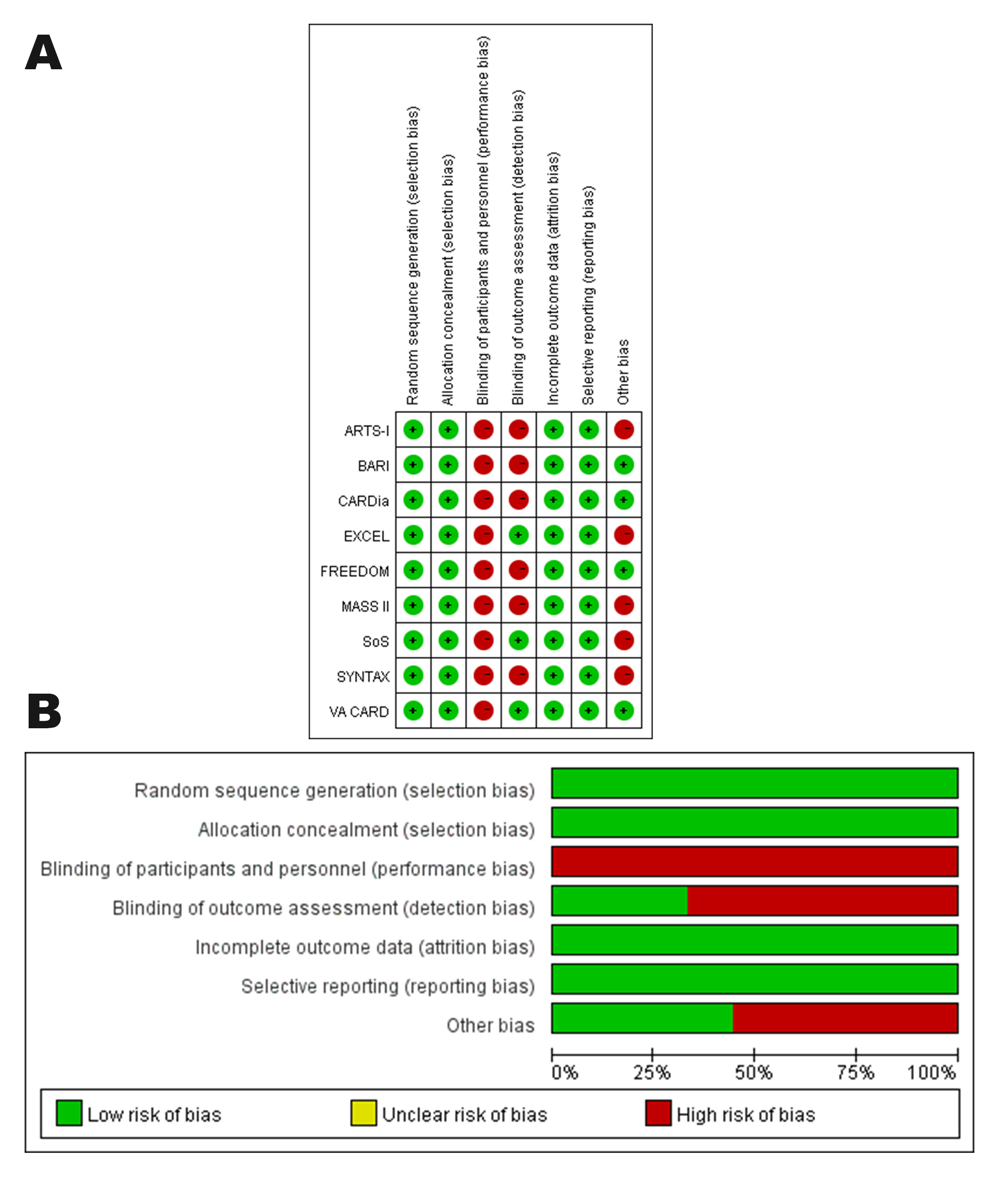

Supplement: Supplementary file 3 — Figure S2 Methodological quality of RCTs included in the meta‐analysis. [file CLC-44-899-s003.tif]
